# Nuclear Nox4-Derived Reactive Oxygen Species in Myelodysplastic Syndromes

**DOI:** 10.1155/2014/456937

**Published:** 2014-02-26

**Authors:** Marianna Guida, Tullia Maraldi, Francesca Beretti, Matilde Y. Follo, Lucia Manzoli, Anto De Pol

**Affiliations:** ^1^Department of Surgical, Medical, Dental and Morphological Sciences with interest in Transplant, Oncology and Regenerative Medicine, University of Modena and Reggio Emilia, Via Del Pozzo 71, 41124 Modena, Italy; ^2^Department of Human Anatomic Sciences, University of Bologna, Via Irnerio 48, 40100 Bologna, Italy

## Abstract

A role for intracellular ROS production has been recently implicated in the pathogenesis and progression of a wide variety of neoplasias. ROS sources, such as NAD(P)H oxidase (Nox) complexes, are frequently activated in AML (acute myeloid leukemia) blasts and strongly contribute to their proliferation, survival, and drug resistance. Myelodysplastic syndromes (MDS) comprise a heterogeneous group of disorders characterized by ineffective hematopoiesis, with an increased propensity to develop AML. The molecular basis for MDS progression is unknown, but a key element in MDS disease progression is the genomic instability. NADPH oxidases are now recognized to have specific subcellular localizations, this targeting to specific compartments for localized ROS production. Local Nox-dependent ROS production in the nucleus may contribute to the regulation of redox-dependent cell growth, differentiation, senescence, DNA damage, and apoptosis. We observed that Nox1, 2, and 4 isoforms and p22phox and Rac1 subunits are expressed in MDS/AML cell lines and MDS samples, also in the nuclear fractions. Interestingly, Nox4 interacts with ERK and Akt1 within nuclear speckle domain, suggesting that Nox4 could be involved in regulating gene expression and splicing factor activity. These data contribute to the elucidation of the molecular mechanisms used by nuclear ROS to drive MDS evolution to AML.

## 1. Introduction

The progression of a premalignant condition to a lethal malignancy is thought to involve an accumulation of mutations in genes that regulate cellular proliferation, survival, and differentiation [1, 2]. The myelodysplastic syndromes (MDSs) can be considered as a representative premalignant hematopoietic disorder that can transform to acute myeloid leukemia (AML) [3].

(Da Watson) MDS comprises a group of anemic disorders of uncertain etiology characterized by abnormal cell morphology in the bone marrow (BM) and peripheral blood cytopenias [4]. According to the International Prognostic Scoring System, the patients with MDS can be divided into 4 prognostic categories: low, intermediate I, intermediate II, and high risk [5]. In about one-third of the patients with MDS, the disease transforms into AML, within months to a few years. These patients usually have a high risk disease, including Int II or high-risk MDS [6]. The causative agent(s) for these secondary events is poorly understood. The excess ROS are known to be a genotoxic stress that can induce DNA damage and mutation following ineffective repair of DNA damage [7–10]. Increased levels of ROS have been detected in both AML and chronic myeloid leukemia [11, 12]. With regard to MDS and oxidative stress, several studies have reported that increased levels of ROS or oxidative DNA damage could be detected in hematopoietic cells from MDS patients [13–15].

It has been demonstrated that RAS mutations in myelodysplastic syndromes/myeloproliferative diseases result in ROS production [16]. Moreover, another player of inositide signaling; that is, phosphatidylinositol 3 kinase (PI3K) has been suggested to be involved, via its substrate Akt, in the survival of MDS blasts [17].

NAD(P)H oxidase complexes, as ROS sources, are frequently activated in AML blasts and strongly contribute to proliferation, survival, and drug resistance of these cells [18–20]. In leukemia cells, ROS generated by Nox4, at least in part, transmit survival signals through the Akt-PI3K pathway while their depletion leads to apoptosis [21]. Furthermore, NADPH oxidases are now recognized to have specific subcellular localizations, thus being required for localized ROS production [22]. Various ROS-generating and ROS-degrading systems seem to play an important role in different compartments of the cell. The nucleus itself contains a number of proteins with oxidizable thiols that are essential for transcription, chromatin stability, nuclear protein import and export, and DNA replication and repair [23].

Kuroda et al. demonstrated that the endogenous Nox4 preferentially localizes to the nucleus in human endothelial cells [24]. Thus, local Nox4-dependent ROS production in the nucleus may contribute to regulation of redox-dependent transcription factor and gene expression involved in cell growth, differentiation, senescence, and apoptosis. In fact, many transcription factors, including AP-1, NF-*κ*B, Nrf2, p53, glucocorticoid receptor, and nuclear kinases, such as PKC (Protein kinase C), Akt, ERK2, and PKA (Protein kinase A), are redox sensitive [22, 25].

In spite of these striking observations, most ROS nuclear substrates have so far remained elusive, as well as nuclear Nox4-derived ROS functions. Therefore, the first aim of this study is to determine if Nox4 isoform is present in the nucleus of MDS cells and the specific localization area of Nox4 complex. We therefore used the human cell line MOLM-13, established from AML secondary to myelodysplastic syndrome and the AML cell line THP1 and/or human blasts obtained from patients with MDS. The broad object of this research is to elucidate the role of nuclear Nox-derived ROS in myelodysplastic syndromes. Therefore, Nox4 expression has been down-regulated and ROS decrease in the nucleus has been checked in order to confirm the nuclear localization and activity of NADPH oxidase.

Then, we searched for binding partners in the nucleus, in particular signaling key molecules. To reveal the interactome proteins that reside in nuclear Nox complex in human MDS/AML cell line, coimmunoprecipitation assay has been performed in order to check Nox interactions with nuclear signaling players. Identification of substrates or binding partners of nuclear NAD(P)H oxidases will pave the way to finding new pharmacological treatments.

The data resulting from the present study could contribute to shedding the light on the molecular mechanisms used by this key intracellular pathway to drive MDS evolution to AML and, in general, in hematological dysfunctions.

## 2. Materials and Methods

### 2.1. Patient Characteristics

Peripheral blood samples (PBMCs) came from 10 MDS patients and 3 healthy normal volunteers who had given informed consent according to the Declaration of Helsinki. The samples came from the Department of Hematology and Medical Oncology (L. e A. Seràgnoli) of the Policlinico S. Orsola, Bologna, Italy. In all of the subjects participating in this study, MDS diagnosis was defined according to WHO classification [26]. For *in vitro* experiments, PBMCs were isolated by Ficoll-Paque (Amersham Biosciences, Sunnyvale, CA, USA) density-gradient centrifugation, according to the manufacturer's protocol.

### 2.2. Cell Culture

The MDS cell line MOLM13 and the AML cell line THP1 were purchased from DSMZ (German Resource Centre for Biological Material). MOLM-13 cells express FLT3-ITD and have been derived from the peripheral blood of a patient with post-MDS AML [27, 28]. MOLM-13 carries internal tandem duplication of FLT3. THP1 is an acute monocytic leukemia cell line.

Cell lines were cultured with 5% CO_2_ at 37°C in RPMI (Mediatech, Inc., Herndon, VA) with 10% fetal calf serum (FCS) and supplemented with 2 mM L-glutamine, 100 U/mL penicillin, and 100 *μ*g/mL streptomycin (all from EuroClone Spa, Italy).

### 2.3. Nox4 Silencing

Retroviral supernatants were produced according to HuSH shRNA Plasmid Panels (29-mer) Application Guide; AM12 cells were transfected with an empty vector (pRS Vector, TR20003), a scrambled vector (HuSH 29-mer non effective pRS vector, TR30012), and four NOX4 gene specific shRNA expression pRS vectors (TI311637, TI311638, TI311639, and TI311640) for 48 h. Retroviral supernatants were then centrifuged at 2000 ×g for 5 minutes and used for target cells (THP1) infection. Where indicated, cells were infected with NOX4 shRNA retroviral vectors, empty vector, or scrambled vector. Forty-eight hours after infection, cells were exposed to 2 *μ*g/mL puromycin (Sigma Aldrich) for 24 hours, and subjected to evaluation of Nox4 expression by Western blotting and confocal analysis and detection of intracellular ROS levels.

### 2.4. Preparation of Cell Extracts

Cell extracts were obtained as described by Maraldi et al. [29]. Briefly, cells were extracted by addition of AT lysis buffer (20 mM Tris-Cl, pH 7.0; 1% Nonidet P-40; 150 mM NaCl; 10% glycerol; 10 mM EDTA; 20 mM NaF; 5 mM sodium pyrophosphate; and 1 mM Na3VO4) and freshly added Sigma Aldrich Protease Inhibitor Cocktail at 4°C for 30 min. Lysates were sonicated, cleared by centrifugation, and immediately boiled in SDS sample buffer or used for immunoprecipitation experiments, as described below.

### 2.5. Nuclei Purification

Cell nuclei were purified as reported by Cenni et al. [30]. Briefly, 400 *μ*L of nuclear isolation buffer (10 mM Tris-HCl, pH 7.8, 1% Nonidet P-40, 10 mM *β*-mercaptoethanol, 0.5 mM phenylmethylsulfonyl fluoride, 1 *μ*g/mL aprotinin and leupeptin, and 5 mM NaF) was added to 5 × 10^6^ cells for 8 min on ice. MilliQ water (400 *μ*L) was then added to swell cells for 3 min. Cells were sheared by passages through a 22-gauge needle. Nuclei were recovered by centrifugation at 400 ×g at 4°C for 6 min and washed once in 400 *μ*L of washing buffer (10 mM Tris-HCl, pH 7.4, and 2 mM MgCl_2_, plus inhibitors as described earlier in the text). Supernatants (containing the cytosolic fractions) were further centrifuged for 30 min at 4000 ×g. Isolated nuclear and cytoplasmic extracts were finally lysed in AT lysis buffer, sonicated, and cleared by centrifugation.

### 2.6. Immunoprecipitation and Electrophoresis

Immunoprecipitation was performed as reported by Bertacchini et al. [31]. For preclearing procedure nuclear lysates were incubated with 2 *μ*g anti-M2 for 1 hour (Sigma Aldrich) and then with beads for additionally 30 min, which were then removed and discarded prior to the immunoprecipitation. Precleared lysates, whose protein concentration was determined by the Bradford method, were incubated 4 hours with 3 *μ*g of anti-Nox4 (Novus Biologicals, CO, USA). Then samples were treated with 30 *μ*L of 50% (v/v) of protein A/G agarose slurry (GE Healthcare Biosciences, Uppsala, Sweden) at 4°C with gentle rocking for 1 h. Pellets were washed twice with 20 mM Tris-Cl, pH 7.0; 1% Nonidet P-40; 150 mM NaCl; 10% glycerol; 10 mM EDTA; 20 mM NaF; 5 mM sodium pyrophosphate, once with 10 mM Tris-Cl, pH 7.4, boiled in SDS sample buffer, and centrifuged. Supernatants were loaded onto SDS-polyacrylamide gel, blotted on Immobilon-P membranes (Millipore, Waltham, MA, USA), and processed by Western blot with the indicated antibodies.

### 2.7. Western Blot

The protocols of the Western blot were performed as described by Hanson et al. [32]. Briefly, protein extracts, quantified by a Bradford Protein Assay (Bio-Rad Laboratories, CA, USA), underwent SDS-polyacrylamide gel electrophoresis and were transferred to Immobilon-P membranes. The following antibodies were used: rabbit anti-ERK1/2, goat anti-Matrin3, goat anti-*β*actin, anti-p22phox (Santa Cruz Biotechnology, Santa Cruz, CA, USA) diluted 1 : 500, rabbit anti-Akt1, rabbit anti-Rac1, and rabbit anti-ERK1/2 (Cell Signalling Technology, Beverly, MA, USA), mouse anti-tubulin, rabbit anti-Nox1, and mouse anti-sc-35 (Sigma Aldrich St. Louis, MO, USA), rabbit anti-Nox4 (Novus Biologicals, CO, USA), rabbit anti-Nox2, and mouse anti-pH2A(Ser139) (Millipore, Billerica, MA, USA) diluted 1 : 1000; peroxidase-labelled anti-rabbit, mouse and goat secondary antibodies diluted 1 : 3000 (Pierce Antibodies, Thermo Scientific; Rockford, IL, USA). Ab dilution was performed in TBS-T pH 7.6 containing 3% BSA. The membranes were visualized using Supersignal substrate chemiluminescence detection kit (Pierce, Rockford, IL, USA). Anti-*β*actin antibody was used as control of protein loading. Quantization of the signal was obtained by chemiluminescence detection on a Kodak Image Station 440CF and analysis with the Kodak 1D Image software.

### 2.8. Confocal Microscopy

Cells were fixed for 20 min in 4% ice-cold paraformaldehyde and then permeabilized with 0.1% Triton X-100 in ice-cold phosphate-buffered saline (PBS) for 5 min. Permeabilized samples were then blocked with 3% of bovine serum albumin (BSA) in PBS for 30 min at room temperature and incubated with primary antibodies (Abs): rabbit anti-Nox4 (Santa Cruz, CA, USA) (diluted 1 : 50), mouse anti-sc-35 (Sigma Aldrich St. Louis, MO, USA) and mouse anti pH2A (Ser139) (Millipore, Billerica, MA, USA) (diluted 1 : 100), in PBS containing 3% BSA for 1 h at RT. Secondary antibody was diluted 1 : 200 in PBS containing 3% BSA (goat anti-mouse Alexa 647 and goat anti-rabbit Alexa 488). After washing in PBS, samples were stained with 1 *μ*g/mL DAPI in H_2_O for 1 min and then mounted with antifading medium (0.21 M DABCO and 90% glycerol in 0.02 M Tris, pH 8.0). Negative controls consisted of samples not incubated with the primary antibody, but only with the secondary antibody.

Confocal imaging was performed on a Nikon A1 confocal laser scanning microscope as previously described [33].

Spectral analysis was carried out to exclude overlapping between two signals or the influence of autofluorescence background on the fluorochrome signals, as previously shown [34]. The confocal serial sections were processed with Image J software to obtain three-dimensional projections, as previously described [35]. The image rendering was performed by Adobe Photoshop software.

### 2.9. Nuclear ROS Imaging

Nuclear ROS were detected with nuclear-localized fluorescent probe for H_2_O_2_, Nuclear Peroxy Emerald 1 (NucPE1) [36–39]. For all experiments, 5 *μ*M solutions of NucPE1 (from 5 mM stocks in DMSO) were made in PBS/glucose. The cells were then kept in an incubator (37°C, 5% CO_2_) for a total of 30 min in the dark. Fluorescence was measured on a multiwell plate reader (Appliskan, Thermo Scientific) using 488 nm filter for excitation and 535 nm filter for emission.

Confocal fluorescence imaging studies were performed with a Nikon A1 confocal laser scanning microscope. Excitation of NucPE1-loaded cells at 488 nm was carried out with an Ar laser and emission was collected at 535 nm. All images in an experiment were collected simultaneously using identical microscope settings. Image analysis was performed in Image J.

### 2.10. Statistical Analysis

*In vitro* experiments were performed in triplicate. For quantitative comparisons, values were reported as mean ± SD based on triplicate analysis for each sample. To test the significance of observed differences between the study groups, unpaired Student's *t*-test was applied. A *P* value <0.05 was considered to be statistically significant.

## 3. Results

### 3.1. Patient Characteristics

Peripheral blood (PB) MCs from 10 patients affected by MDS (5 treated with azacitidine, 2 with hydroxyurea, 1 with erythropoietin and 2 with best supportive care only) were examined. Median age was 70 years (range 65 to 82 years). MDS was diagnosed following World Health Organization (WHO) classification [26]. Patient demographics and disease characteristics are summarized in Table 1. Karyotype analysis shows that different abnormalities are present in the study group as well as different disease gravity levels, as shown by WHO classification.

### 3.2. NADPH Oxidases Expression in MDS Samples and MDS/AML Cell Lines

At first, we tested the expression level of NADPH oxidase isoforms and their subunits by Western blot (WB) analysis of total lysates of all the MDS collected samples and of human MDS/AML cell lines (THP1 and MOLM-13).

**Figure 1:**
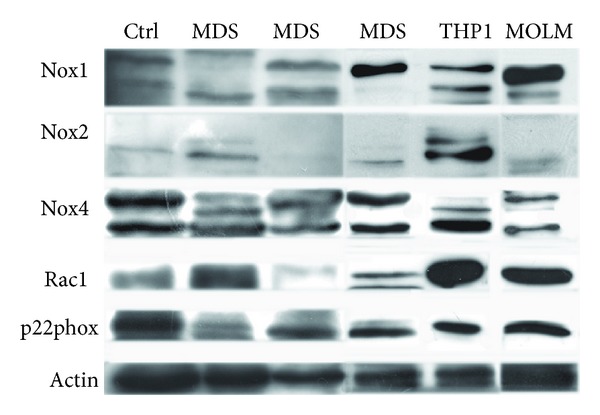
Expression of NADPH oxidases and their subunits. Representative images of Western blot analysis of total lysates of PBMC healthy donor (ctrl), MDS, MOLM-13, and THP1 samples revealed with antibodies against NADPH oxidase isoforms 1, 2, and 4, Rac1, and p22phox subunits. *β*actin was used as loading internal control.

By using different kinds of affinity-purified antibodies raised against distinct immunogens from human Nox1, Nox2, Nox4, p22phox, and Rac1, we demonstrated that all these proteins are present in MDS samples and human MDS/AML cell lines.  Figure 1 shows the expression pattern of three representative MDS samples compared to MOLM-13 and THP1 cell lines and PBMC healthy donor. Nox1 and Nox4 isoforms seem to be highly expressed in all samples.

**Figure 2:**
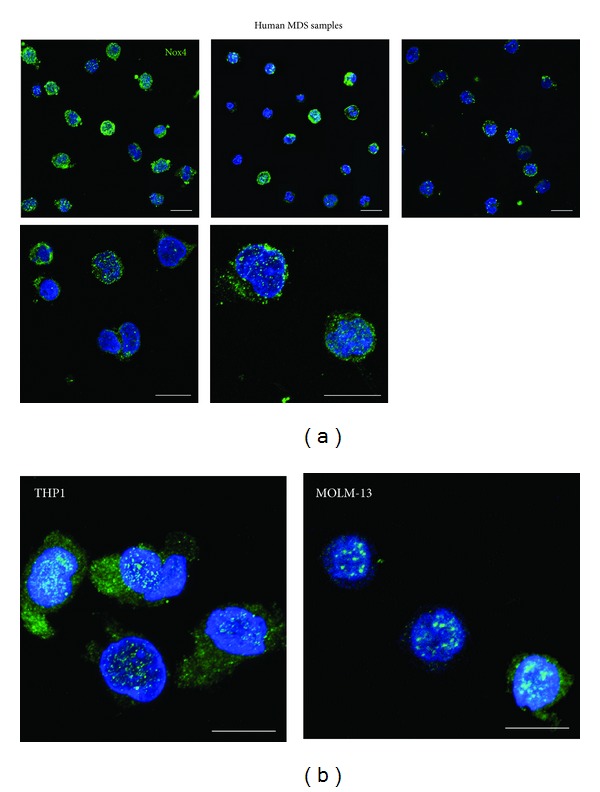
Immunofluorescence analysis of Nox4 expression. (a) Representative images at different magnifications showing superimposing between DAPI (blue) and Nox4 (green) signals in three human MDS samples (from left to right RAEB1, RAEB2, and RARS first row and RAEB1 and RAEB2 in the second row). (b) Representative images showing superimposing between DAPI (blue) and Nox4 (green) signals in MOLM-13 and THP1 samples. Scale bar: 10 *μ*m.

Interestingly, Nox4 is both expressed into the nucleus and in the cytoplasm. In fact, confocal analysis (Figure 2(a)) demonstrated that in different MDS samples (images representative of RAEB1, RAEB2, and RARS are shown) a punctate staining of Nox4 is detectable inside the nuclei. Nox1 and Nox2 signals show a cytoplasmic localization (not shown). The same pattern has been observed also in MDS/AML cell lines (Figure 2(b)).

**Figure 3:**
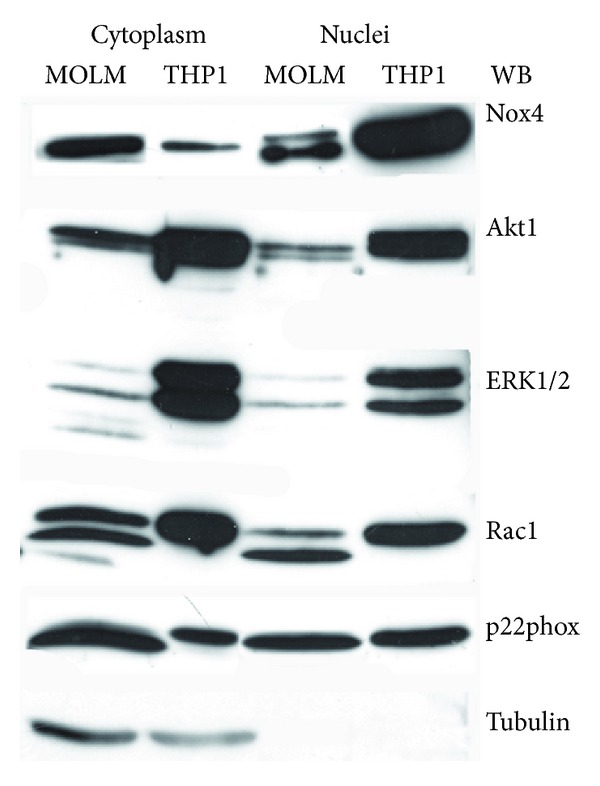
Western blot analysis of cytosol (cyto) and nuclear fractions (nuclei) of MOLM-13 and THP1 samples revealed with anti-Nox4, anti-Akt1, anti-ERK1/2, anti-Rac1, and anti-p22phox. Tubulin absence was used as index of nuclear extract's purification. Presented data are representative of three independent experiments.

In order to demonstrate the specificity of the immunofluorescence signal, we performed Western blot analysis of nuclear and cytoplasm subfractions (Figure 3). Also with this approach we can see a high presence of Nox4 in nuclear portions; moreover, the Nox4 subunit p22phox is present in both the subfractions.

Rac, an important downstream effector of RAS, is an activator of Nox2 and Nox4 and, in leukemic cells, Rac-1 and Akt activate Nox2 and Nox4 [20, 40]. RAS/Nox have been also demonstrated to be modulators of cell growth and proliferation via activating the mitogen-activated protein kinase ERK1/2 signaling pathway [41]. Beside Nox4 and its regulators p22phox and Rac-1, here we show that Akt1 and ERK1/2 are also present in nuclear protein portions of both MOLM-13 and THP1 cell lines (Figure 3).

### 3.3. Modulation of Nox-Derived Nuclear ROS Production

**Figure 4:**
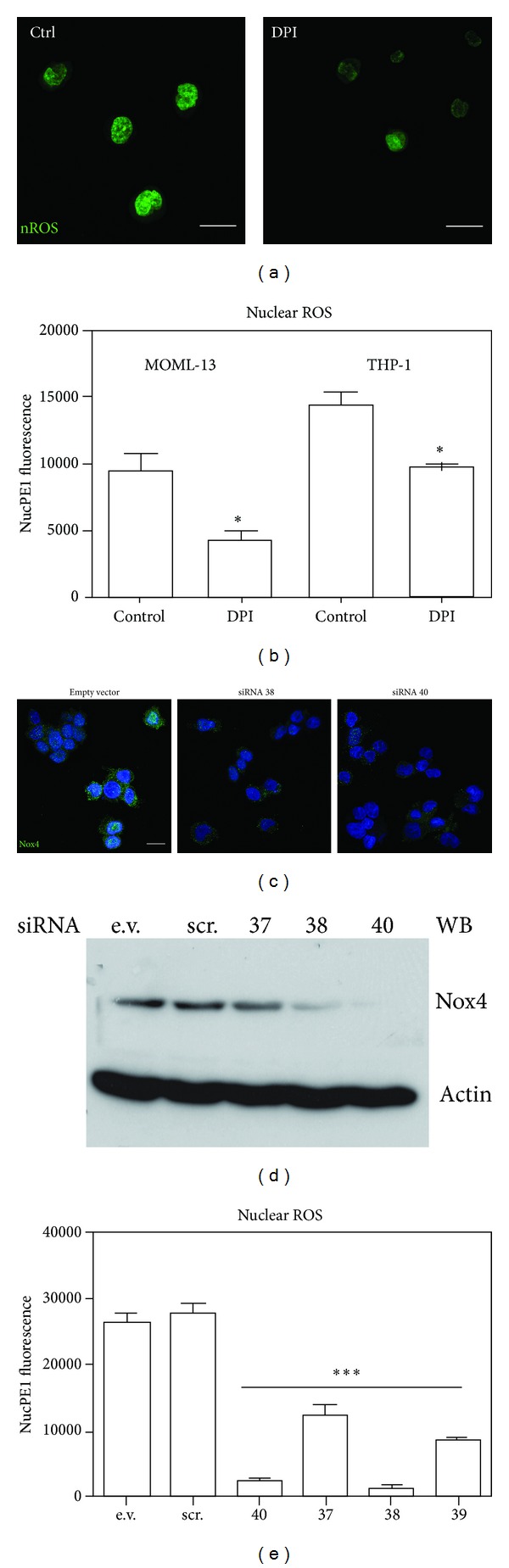
Effect of Nox4 inhibition on nuclear ROS production. (a) Representative images showing staining with nuclear ROS probe (nuclear peroxy Emerald 1) of THP1 in the presence or absence of 2 *μ*M DPI for 18 hours. Scale bar: 10 *μ*m. (b) Graph representing fluorescence intensity of nuclear ROS probe (Nuclear peroxy Emerald 1) of MOLM-13 and THP1 in the presence or absence of 2 *μ*M DPI for 18 hours. (c) Representative images showing: superimposing between DAPI (blue) and Nox4 SC (green) signals of THP1 treated with empty vector (EV) or Nox4-directed siRNA (38 and 40) as reported in Section 2. Scale bar: 10 *μ*m. (d) Representative images of Western blot analysis of Nox4 silencing in THP1 cells. *β*actin was used as loading internal control. (e) Graph representing fluorescence intensity of nuclear ROS probe (Nuclear peroxy Emerald 1) of THP1 treated with empty vector (EV), scrambled siRNA (SCR), or Nox4-directed siRNA (37, 38, 39, and 40). Presented data are representative of three independent experiments. *P < 0.05 and ***P < 0.001 versus Control.

In order to investigate the NADPH oxidase activity inside the nuclei, we used a nuclear selective probe for H_2_O_2_, Nuclear peroxy Emerald 1 (Figure 4). Confocal microscopy confirms that there is a ROS production inside the nuclei (Figure 4(a)).

Even if the use of Nox4 synthetic inhibitor, diphenyleneiodonium (DPI), is not directed to the nuclear part of Nox4, as demonstrated by the fluorogenic probe assay, the Nox4 activity inhibition reduces the nuclear ROS production (Figures 4(a) and 4(b)).

A more selective approach, as Nox4 silencing, confirms the Nox4 role in nuclear ROS production. The highest downregulation of Nox4 was obtained with shRNA TI311638 and TI311640, as demonstrated by Western blot (Figure 4(d)). Immunofluorescence assay (Figure 4(c)) shows that the decrease in Nox4 expression occurs both in cytoplasmic and nuclear compartments. Overall, THP1 cells, treated with all shRNA sequences, show a significant decrease in nuclear ROS level.

### 3.4. Nuclear Nox4 Role

The production of ROS directly inside the nuclei can be linked to DNA damage. In fact, increasing evidence suggests that genetic changes in myeloid malignancies lead to increased production of endogenous sources of DNA damage, such as reactive oxygen species.

It has been shown recently that the phosphorylation level of H2AX is crucial to determining whether cells will survive after DNA damage [42].

**Figure 5:**
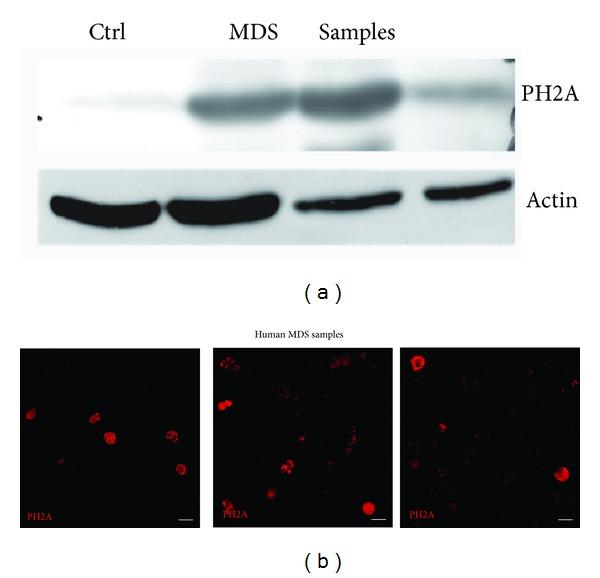
DNA damage in MDS samples. (a) Representative images showing staining with anti-PH2A (red), as marker of DNA damage, in three human MDS samples. Scale bar: 10 *μ*m. (b) Western blot analysis of total lysates of MDS samples revealed with anti-PH2A. *β*actin was used as loading internal control.

Looking at nuclear H2A foci, as expected, we found that, compared to healthy donor, MDS samples exhibit a huge status of H2A phosphorylation (Figure 5), suggesting that nNox4-generated ROS can induce nuclear DNA damage.

**Figure 6:**
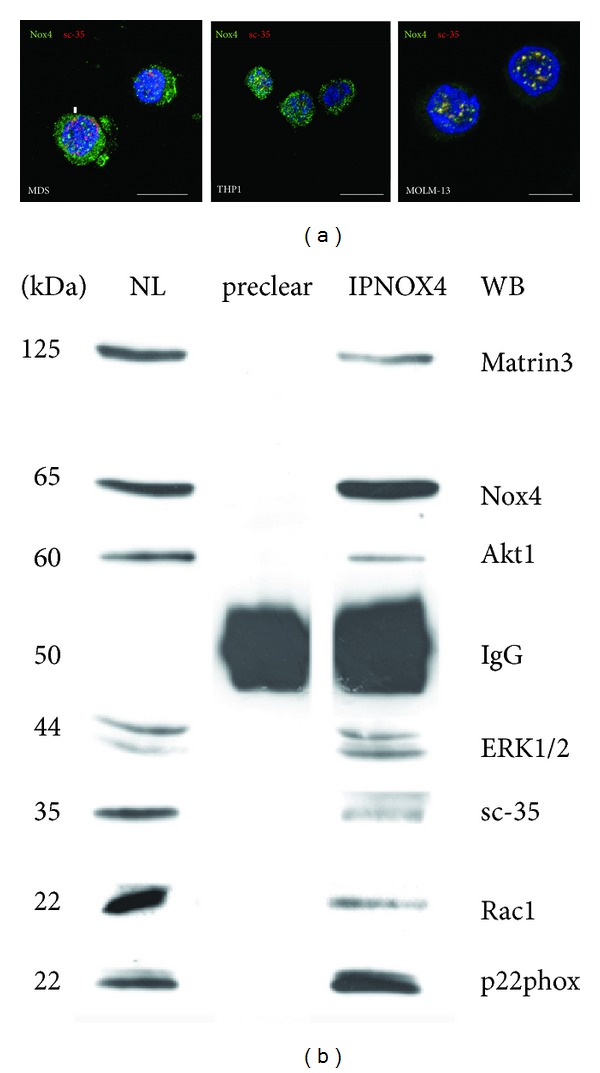
Nox4 nuclear interaction. (a) Representative images showing superimposing between DAPI (blue), Nox4 (green), and sc-35 (red) signals in MDS, MOLM-13, and THP1 cells. Scale bar: 10 *μ*m. (b) Representative images of Western blot analysis of nuclear lysate (NL), preclearing (preclear) sample obtained, as described in methods section, before immunoprecipitation experiment with Nox4 antibody (IPNOX4): these samples were then revealed with anti-Matrin3, anti-Nox4, anti-Akt1, anti-ERK1/2, anti-sc-35, anti-Rac1, and anti-p22phox. All presented data are representative of three independent experiments.

Then, we investigated the nuclear Nox4 binding network (Figure 6). Based on the punctate Nox4 signal previously observed (Figure 2), we tested whether this distribution follows the localization of speckles nuclear domains by using an antibody directed against sc-35. Sc-35 is involved in pre-mRNA splicing and is found in the bodies in the nucleus referred to as speckles, sc-35 domains, or splicing factor compartments (SFCs).  Figure 6(a) shows that the nuclear signal of Nox4 (green) often colocalizes with the one of sc-35 (red), generating an orange staining. The arrow indicates, as example, the colocalization in MDS sample.

The interaction of Nox4 with sc-35 was confirmed also by coimmunoprecipitation experiment (Figure 6(b)). Nuclear extracts (NL) of THP1 were used for coimmunoprecipitation analysis with anti-Nox4 (IPNox4), since this cell line express the highest level of nuclear Nox4. Preclearing fraction, obtained as described in method section, is shown as control for nonspecific interactions with protein A/G: the only one band is the one of IgG used in the preclearing step. This experiment also confirms the interaction between Nox4 and the subunit p22phox. Furthermore, Nox4 seems to be linked with nuclear matrix protein, Matrin3, and with ERK1/2 and Akt1, suggesting a direct role in nuclear MAPK and Akt signaling regulation.

## 4. Discussion

Myelodysplastic syndromes refer to a heterogeneous group of closely related hematological disorders that are characterized by an ineffective production of blood cells (dysplasia) and a hypercellular or hypocellular marrow with impaired morphology and maturation (dysmyelopoiesis) [5]. Although the genetic basis of MDS is not completely understood, a significant percentage of MDS cases are characterized by chromosomal aberrations [43, 44] and the transformation of MDS to AML is often accompanied by additional mutations [45]. Approximately, 30% myelodysplastic syndrome (MDS) cases progress to acute myelogenous leukemia.

It is now well established that the progression of normal cells to neoplastic transformation results from the accumulation of mutations in genes that control cellular proliferation, survival, and differentiation [1].

It has been proposed that AML requires a minimum of two complementary mutations, one leading to enhanced proliferation and the second leading to impaired differentiation [46].

A significant percentage of MDS cases is characterized by chromosomal deletions of 5q or 7q [3] and has previously been reported to be high in a proportion of myeloid malignancies [47, 48]. The next most frequent genetic alteration in MDS is activating mutations of the RAS homologues occurring in 20% of MDS patients reviewed in [49, 50]. Tumor progression is accompanied by an increase in ROS, which leads to an increased DNA damage. It is well established that activation of oncogenes can lead to ROS production [51], and ROS is an established source of endogenous double-strand breaks [52]. Thus, acquisition of oncogenic changes can initiate a cycle of genomic instability that has the potential to create further mutations, which in turn may facilitate leukemic disease progression. Several lines of evidence now indicate that activation of RAS-mitogen-activated protein (MAP) kinase pathways can generate increased ROS. In fact, one candidate pathway for ROS production in MDS may be signaling through RAC1 [53]. Another candidate pathway for ROS production is signaling through extracellular signal-regulated kinase 1/2 (ERK1/2).

It has been previously demonstrated that ERK phosphorylation occurred downstream from the Nox4 pathway, but through the RAS activation in endoplasmic reticulum [54]. The presence and the activity in the nucleus of both PI3K/Akt [55] and NAD(P)H oxidase isoform 4 have been described [24]. Indeed, the altered expression of Nox4 could be involved in a dysregulation of cell cycle and has also an important meaning in high risk MDS patients. ROS can inactivate nuclear-localized phosphatases and thereby enhance kinase activation. Moreover, excessive production of ROS also could lead to oxidative DNA damage.

In this point of view, the subcellular localization of Nox4 is likely to be especially important, given its constitutive activity, unlike isoforms, such as Nox1 or Nox2, that require agonist activation.

We observed in human MDS samples, showing DNA damage sign and obtained from different disease grade patients, that Nox4 isoform is, interestingly, localized into the nucleus. Inhibition of Nox4 activity, obtained with DPI or Nox4 silencing, induces a decline of nuclear ROS production, confirming the activity of Nox4 within the nuclei.

Confocal and coimmunoprecipitation analysis demonstrate Nox4 presences in speckle domains suggesting that Nox4 could be involved in regulating DNA-mRNA processing machinery by ROS production in specific nuclear area. Also Matrin 3 has been demonstrated to bind DNA at sites termed scaffold/matrix attachment regions to regulate gene expression through interactions with chromatin remodeling [56]. Here, we show that Nox4 coimmunoprecipitates also with Matrin 3. Thus, Matrin 3 could be a docking site where nuclear ROS signaling may exert its function on transcription/pre-mRNA modulation in specific nuclear domains.

Moreover, immunoprecipitation analysis demonstrated that Nox4 interacts with Akt and ERK signaling, suggesting a role in nuclear signaling dysregulation leading to MDS progression. The identification of these binding partners of nuclear Nox4 may pave the way to finding new pharmacological treatments.

Taken together, we suggest that nNox4 regulation may have important pathophysiologic effects in MDS through modulation of nuclear signaling and DNA damage. For example, Nox4 can be a critical mediator in oncogenic RAS-induced DNA-damage response.

In addition to antioxidant therapy, targeted therapy for STAT, RAS, and PI3K pathways, such as RAC1 [16], may be amenable to inhibition of nuclear ROS sources and genomic instability using small molecule inhibitors.

These therapeutic options are likely to represent important treatments in MDS/AML. Nevertheless, efficacy of ROS reduction on the reversal of genomic instability and disease progression may rely on elucidation of the major routes for ROS overproduction in cancer with multiple genetic alterations.

## Figures and Tables

**Table 1:** Clinical, hematologic, and cytogenetic characteristics of MDS patients.

WHO diagnosis	Karyotype	Treatment	Clinical outcome
RARS	Normal		Stable disease
RCMD	del (7q)		Disease progression, death
RCMD	del (20q)	EPO	AML
RAEB1	Normal	5-aza	RAEB2
RAEB1	del (5q)	Idrossiurea (HU)	Stable disease
RAEB1	del (7q)	Idrossiurea (HU)	Stable disease
RAEB2	Tris (8)	5-aza	AML, death
RAEB2	Tris (8)	5-aza	AML, death
RAEB2	Normal	5-aza	AML, death
AML	Normal	5-aza	AML, death

## Abbreviations

AML: acute myeloid leukemia
BM: bone marrow
BSA: bovine serum albumin
DABCO: 1,4-diazabicyclo(2.2.2)octane
DAPI: 4′,6-diamidino-2-phenylindole
DPI: diphenyleneiodonium
EDTA: ethylenediaminetetraacetic acid
PBMCs: peripheral blood samples
PBS: phosphate buffered saline
MDSs: myelodysplastic syndromes
Nox: NADPH oxidase
nNox4: nuclear NOX4
RA: refractory anemia
PI3K: phosphatidylinositol 3 kinase
RARS: RA with ringed sideroblasts
RAEB: RA with excess of blasts
RCMD: refractory cytopenia with multilineage dysplasia
ROS: reactive oxygen species
TBS: Tris-buffered saline
Tx: Triton-X-100.
